# Impact of Guaranteed Income on Health, Finances, and Agency: Findings from the Stockton Randomized Controlled Trial

**DOI:** 10.1007/s11524-023-00723-0

**Published:** 2023-04-10

**Authors:** Stacia West, Amy Castro

**Affiliations:** 1 The University of Tennessee College of Social Work, Co-Founding Director, The Center for Guaranteed Income Research at the University of Pennsylvania, Philadelphia, USA; 2 The University of Pennsylvania, School of Social Policy and Practice, Co-Founding Director, The Center for Guaranteed Income Research, Philadelphia, USA

**Keywords:** Guaranteed income, Universal basic income, Cash transfers, Mental health

## Abstract

The purpose of this experiment was to test the effects of a $500 per month guaranteed income for 2 years on health and financial outcomes. A mixed-methods randomized controlled trial in Stockton, CA, USA enrolled 131 individuals to the treatment condition and 200 to control to receive a guaranteed income from February 2019 to January 2021. Quantitative data collection began 3 months prior to allocation at 6-month intervals concluding 6 months after withdrawal of the intervention. Qualitative data collection included 105 interviews across 3 stages. The primary outcomes were income volatility, physical and mental health, agency, and financial wellbeing. The treatment condition reported lower rates of income volatility than control, lower mental distress, better energy and physical functioning, greater agency to explore new opportunities related to employment and caregiving, and better ability to weather pandemic–related financial volatility. Thus, this study provides causal evidence of positive health and financial outcomes for recipients of guaranteed income. As income volatility is related to poor health outcomes, provision of a guaranteed income is a potentially powerful public health intervention.

**Supplementary Information:**

The online version contains supplementary material available at 10.1007/s11524-023-00723-0.

In 2017, Basu characterized income volatility as a public health threat [1]. Prior to that, income volatility—month over month increases or decreases to average income—were most severe among very low-income households [2]. Negative impacts of income volatility include incidence of cardiovascular disease [3], depression and anxiety [4], and cognitive decline [5]. Income volatility reached unprecedented levels under COVID. The monthly poverty rate hovered between 11.2 and 12% for white households; for Black and Hispanic families, it was double [6]. Pandemic-related income volatility forced impossible choices between limiting virus exposure, basic needs, and health care costs [7].

Empirical evidence from behavioral economics and public health demonstrate that the constant experience of scarcity generates less competence, coping, and a reduced cognitive capacity for decision-making, which can exacerbate financial fragility and uncertain employment conditions [8, 9]. Furthermore, this persistent volatility generates negative health outcomes [10–12]. Thus, if income volatility produces poor health, may guaranteed income (GI) mitigate it? Positive impacts of unconditional cash include reductions in hospitalization [13], rates of low birth weight [14], food insecurity [15], and incidences of psychiatric disorders [16]. After the negative income tax experiments between 1968 and 1980, no research was conducted on guaranteed income in the USA, likely due to misinterpretation of findings hinting at a negative impact of unconditional cash on labor supply [17] and shifts toward neoliberal policies emphasizing benefits reduction [18].

Given few empirical priors to theoretically ground a pathway of change for guaranteed income, we ground our hypotheses on the counterfactual. Receipt of a consistent unconditional monthly cash payment should mitigate income volatility and some financial stress. Reduction of some financial stress should allow recipients to meet basic needs and weather unforeseen financial shocks more easily. The ability to do so should lower psychological and emotional distress, freeing up a person’s cognitive pathways to imagine and pursue new opportunities. The Stockton Economic Empowerment Demonstration (SEED) launched in 2018. SEED provided a monthly unconditional cash transfer, or GI, of $500 for 24 months to answer: How does GI impact monthly income volatility? To what degree does a GI impact psychological distress, and physical functioning? How does GI generate agency over one’s future? How were financial wellbeing and agency attenuated by the pandemic?

## Methods

Our approach included two strands: (1) a staged parallel, sequential strand [19] to integrate findings at two points in the experiment (*quant* + *qual–* > *meta-inference –* > *quant* + *qual–* > *meta-inference)*, and (2) community-based participatory research (CBPR) with Stocktonians outside SEED. Stage one encompassed year one and stage two the second with additional COVID questions. The CBPR research activities and qualitative data on secondary outcomes focus on sub-strands beyond this paper’s scope.

### Sampling and Randomization

Funding supported 131 individuals in treatment over 2 years. A control group 200 was indicated for estimated attrition of 20% to produce a conservative minimum detectable effect. With a non-directional hypothesis, with power set at 0.80 and alpha set at 0.05, MDE was *f* = 0.30. We note that both the small sample size allowed due to the cost of the intervention, as well as a lack of priors on effect sizes of guaranteed income interventions on various outcomes limits specificity of the power analysis.

Recruitment used a stratified random sample of households within census tracts at or below Stockton’s household area median income of $46,033. Forty-two census tracts meeting criteria were selected, and Delivery Sequence File (DSF) lists were purchased from a licensed vendor. A percentage of addresses was drawn from each tract based on population proportion. A mailer to participate in SEED and research was sent to 4200 households. Mailers were addressed to households allowing anyone to respond thereby assisting in mitigating benefits loss [20]. Mailers included a Qualtrics link for the baseline, with a consent form on the opening page. Consented participants were randomized, using simple random assignment in Stata with allocation concealment. The research team generated the random allocation sequence and assignment. SEED staff enrolled participants into treatment.

### Quantitative Measurement and Analysis

Data collection began in December 2018. Subsequent waves were as follows: Wave 2: January/February 2019; Wave 3: August 2019; Wave 4: February 2020; Wave 5: August 2020; Wave 6: February 2021 (Final disbursement); and Wave 7: August 2021 (6-month follow-up). The onset of the pandemic in March 2020 directed analytic decision-making, as the history effect threatened both the internal and external validity of the experiment. As such, quantitative data were separated to test the effects of guaranteed income given typical economic, environmental, and health threats as well as the withdrawal of the intervention. As such, the data were analyzed at three time points: baseline of December 2018 through Wave 4 of February 2020, Wave 5 of August 2020 through Wave 6 in February 2021 to capture data during the first year of the pandemic, Wave 7 in August 2021 to test effects of the withdrawal of the intervention.

#### Primary outcomes

Income volatility was measured monthly through self-reporting via SMS. Only values listed as $0 per month in the treatment group were imputed at $500 to reflect the receipt of the guaranteed income. Income volatility was calculated by the coefficient of variation, and can be understood as the month over month swing in income. For example, if a person earns $2000 per month, and their coefficient of variation in income over the observation period is 0.25, they experience an average monthly change in income of $500. One-tailed *t*-tests at each time point (baseline to 1 year and second year) were conducted. Physical functioning and psychological distress were measured, respectively, every 6 months via the Short Form Health Survey-36 [21] (SF-36) and the Kessler 10 [22]. Both are widely used instruments to measure self-reported physical and emotional health in clinical and survey research settings with diverse populations. These outcomes were scored and analyzed using ANCOVA at the following time points: baseline to 1 year, second year, and 6 months after withdrawal of the guaranteed income.

#### Secondary outcomes

Financial effects of the pandemic were measured through the Consumer Financial Protection Bureau’s Financial Wellbeing Scale (FWS), administered at 6-month intervals beginning at Wave 5 [23]. The scale was scored and analyzed only for Waves 5 through 6 and 6 through 7, as it was added as part of additional inquiry of financial conditions associated with the pandemic. ANCOVA was used to conduct these analyses. Financial wellbeing was also measured and the commonly asked question: suppose that you have an emergency expense that costs $400. Based on your current financial situation, how would you pay for this expense? This was administered at 6-month intervals beginning at Wave 2 [24], and analyzed from Waves 2 to 4, 4 to 6, and 6 to 7 using ANCOVA. This variable was recoded prior to analyses to create a binary outcome, whereby 0 = could not afford a $400 emergency (pay using debt, pay by borrowing from a friend or family member, pay by selling something, or I could not pay) and 1 = could afford a $400 emergency (pay using case, pay using a credit card that would be paid off in full.

Agency was measured by change in employment status from baseline to Wave 4, 4 to 6, and 6 to 7 using ANCOVA. Employment status was shifted from a categorical to binary variable and coded as 1 = eligible for employment and employed (full-time employed, part-time employed, stay-at-home parent or caregiver) or 0 = eligible for employment but not employed (unemployed and looking for work and unemployed and not looking for work). Individuals who indicated they were ineligible for employment due to retirement, disability, or student status were excluded from the analyses.

A key tenant of guaranteed income is unconditionality; thus, even members of the treatment group were not compelled to participate in research activities as a condition of receiving the guaranteed income. Statistical power was limited by attrition and differential outcomes of the politically purposive cohort (*n* = 14) [25] which required exclusion. By endline, retention was approximately 35% in control and 55% in treatment dependent upon outcome measure. Per the pre-analysis plan [26], attrition was not found to be correlated with group assignment, could not be predicted from baseline characteristics, and baseline characteristics of attritors were not different from those in control. Treatment effects were not bounded. While multiple imputation methods could have bolstered statistical power, it was employed due to the conditions of the pre-analysis plan. Analysis proceeded as intention to treat.

### Qualitative Measurement and Analysis

Three stages of semi-structured interviews occurred (*N* = 105). All were digitally recorded, professionally transcribed, and used pseudonyms. The second author designed all protocols and codebooks and supervised five coders. The first author contributed to protocols and conducted interviews. Coding utilized Dedoose.

#### Stage one

The first sample (N = 36) included participants recruited during SEED’s treatment orientation. The 20-min interviews focused on trust, networks, and decision-making. Thematic analysis was conducted on a semantic level using Braun and Clark’s [27] phases with architectural, emotion, and values codes [28].

#### Stage two

Stage one informed protocols for stage two (*N* = 50; n = 35 treatment; *n* = 15 control). This included 1–2-h interviews at year one’s mid-point either at home or in the community. The protocol captured adaptations, strategies, and sense of agency associated with receiving GI through prompts on pooling, deservedness, the safety net, and stressors. Stage one analysis indicated that the $500 was being interpreted as an unfolding phenomenon without concrete language [29], because the cash did not require means testing. Thus, stage two incorporated grounded theory at the latent level alongside thematic analysis on a semantic level with theoretical coding [28], process codes [25], values codes [27], and focus coding [27].

#### Stage three

Stage 3 (*N* = 19; *n* = 5 control; *n* = 14 treatment) occurred during 2020. We planned 60 interviews, but the pandemic altered this. All interviews shifted to zoom but continuing caused an undue burden. Fatigue from shifting one’s entire life online coupled with remote education, lack of privacy, sporadic internet, and wildfire pressures halted qualitative data collection. The remaining interviews addressed (1) take-up, (2) adaptations, (3) perceptions of pandemic interventions vs. GI, and (4) uncertainty. Thematic analysis covered 1–3 and focused on process-coding to determine how beliefs about institutional failures may influence motivations on a semantic level. Item 4 rested on grounded theory by employing theoretical coding alongside focus coding at a latent level. Since the pandemic was collectively and individually experienced, integrating focus and theoretical coding alongside thematic analysis represented an optimal choice because it explicitly surfaces phenomenon experienced by many, but lacks shared understanding and language [29].

## Results

From 4200 invitations mailed, 505 baseline surveys returned; 27 duplicates were removed. Allocation of 478 applications were as follows: 131 to treatment, 200 to control, and 147 to administrative control. By Wave 7, 2 members of control and 7 members of treatment withdrew. One hundred and twenty members of control and 67 members of treatment were lost to follow-up by Wave 7. Intent to treat analyses were conducted for 198 members of the control group and 110 members of the treatment group.

Gender was approximately 70% female and 30% male (Table 1). Nearly half of treatment and control were white, with one-third Black or African American. The treatment had nearly double the representation and Asian and Pacific Islanders than control, and both groups had just over one third Hispanic or Latino. Approximately 75% of participants lived in an under four-person household, and around 50% had children in the household. Most were single (59%), with 40% married or partnered. The average age was 40 years in control and 45 in treatment. Forty percent reported full- or part-time employment. More individuals in treatment were stay at home parents (11%) than control (7%). In both, approximately 75% had at least a high school education or equivalent. The median income of the control group was $1957 compared to $1886 for treatment.

**Table 1:** Descriptive statistics of treatment and control SEED participants at baseline December 2018

	Control (*n* = 198)	Treatment (*n* = 110)
Gender		
Female	68%	69%
Male	32%	30%
Non-conforming	0%	1%
Race		
White	44%	47%
Black/African American	33%	28%
API	7%	13%
Other	17%	12%
Hispanic/Latinx	36%	37%
Household size		
< 4 persons	73%	72%
5–8 persons	24%	25%
> 8 persons	3%	3%
Kids in household	53%	48%
Relationships status		
Single	59%	59%
Partnered	15%	13%
Married	26%	27%
Age		
< 25 years	6%	10%
25–50 years	56%	50%
> 50 years	38%	40%
Employment status		
Disabled	18%	23%
Employed full time	32%	25%
Employed part time	11%	15%
I am a student and do not work	3%	6%
I work seasonally	2%	2%
Retired	10%	6%
Stay at home parent or caregiver	7%	11%
Unemployed looking for work	14%	11%
Unemployed not looking for work	3%	2%
Highest education		
Associate’s degree (2-year college degree)	14%	14%
Bachelor’s degree (4-year college degree)	9%	9%
Elementary school (through grade 5)	2%	0%
GED (diploma equivalency test)	12%	16%
High school diploma	44%	37%
Middle school (6th grade to 9th grade)	3%	2%
No formal education	1%	1%
Other education choice not listed	2%	5%
Other post-graduate degree	3%	5%
Trade or technical school	11%	12%
Monthly income		
Median	$1957	$1886

### Primary outcomes

In year one, the treatment group’s income volatility was 19% compared to control 26%, and was statistically significant (*t* = 1.76, *p* = 0.039). In year two, the treatment group’s income volatility month over month was 22% compared to 25% in control and though the direction followed the one-tailed direction of year one, was not statistically significant. Holding baseline scores constant, Kessler 10 scores, a measure of psychological distress, were lower in treatment rather than control at a significant level from baseline to Wave 4 (*F* = 4.983, *p* = 0.027),but not in the pandemic year or after withdrawal of the intervention. Kessler 10 scores can range from 10 to 50, with higher scores indicating more severe psychological distress. Scores less than 20 indicate a person is likely to be well, and scores 20–24 indicate a mild mental health disorder [22]. Tables 2 and 3 show these between group changes of the treatment group moving from “likely to have a mild mental health disorder” at baseline to “likely to be well” one year into receiving the guaranteed income. This phenomenon is not observed in the control group as the scores hover along the margin of “likely to have a mild mental health disorder” across the three analytic points.

**Table 2:** Descriptive statistics of treatment and control SEED participants for Kessler 10 scores, December 2018 through August 2021

*Model 1: Baseline and covariate adjusted descriptive statistics for Kessler 10 scores at baseline Wave 4, Wave 6, and Wave 7*												
Group	Baseline (December 2018)			Wave 4: February 2020 (adjusted)			Wave 6: February 2021 (adjusted)			Wave 7: August 2021 (adjusted)		
	*N*	Mean	SE	*N*	Mean	SE	*N*	Mean	SE	N	Mean	SE
Control	184	20.8	0.6	88	21.4	0.9	83	23.1	0.9	63	21.0	0.7
Treatment	110	21.3	0.8	87	18.4	0.9	72	20.3	0.8	65	22.0	0.7

**Table 3:** Analysis of covariance (ANCOVA) of treatment and control SEED participants for Kessler 10 scores by group

Model 1: ANCOVA for Kessler 10 scores baseline (December 2018) through Wave 4 (February 2020)						
	Type III sum of squares	df	Mean square	F	Significance	Partial eta squared
Treatment	358.95	1	358.95	4.98	0.027	0.03
Error	12,389.01	172	72.09			
R^2^	0.24					
Model 2: ANCOVA for Kessler 10 scores Wave 4 (February 2020) through 6 (February 2021)						
	Type III sum of squares	df	Mean square	F	Significance	Partial eta squared
Treatment	22.26	1	22.26	0.84	0.360	0.01
Error	3432.51	130	26.40			
R^2^	0.57					
Model 3: ANCOVA for Kessler 10 Sscores Wave 6 (February 2021) through 7 (August 2021)						
	Type III sum of squares	df	Mean square	F	Significance	Partial eta squared
Treatment	9.39	1	9.39	0.34	0.558	0.01
Error	3381.07	124	27.27			
R^2^	0.55					

The SF-36, which measures 8 subscales of mental and physical wellbeing, showed significant between group changes largely in the pre-pandemic year of the experiment (Tables 4 and 5). Holding baseline scores constant, the treatment group was significantly better off at Wave 4 in the following domains than control: pain (*F* = 4.724; p = 0.031); energy over fatigue (*F* = 7.505; *p* = 0.007); emotional wellbeing (*F* = 7.749, *p* = 0.006); role limitations due to emotional health (*F* = 7.052, *p* = 0.009); and physical functioning (*F* = 4.396, *p* = 0.037). During the pandemic, the treatment group indicated better physical functioning (*F* = 0.491, *p* = 0.036) than did control, while holding Wave 4 constant. After withdrawal of the guaranteed income, no between groups effects were detected.

**Table 4:** Descriptive statistics of treatment and control SEED participants for SF-36 subscale scores, December 2018 through August 2021

*Baseline and Covariate Adjusted Descriptive Statistics for SF-36 Subscales Baseline, Wave 4, Wave 6 and Wave 7*													
	Group	Baseline (December 2018)			Wave 4: February 2020 (adjusted)			Wave 6: February 2021 (adjusted)			Wave 7: August 2021 (adjusted)		
General health		*N*	Mean	SD	*N*	Mean	SE	*N*	Mean	SE	*N*	Mean	SE
	Control	107	56.0	2.9	79	56.7	2.1	88	57.1	2.8	70	56.6	2.3
	Treatment	88	54.4	2.7	99	57.8	2.4	67	60.0	3.4	66	56.9	2.4
	Group	Baseline (December 2018)			Wave 4: February 2020 (adjusted)			Wave 6: February 2021 (adjusted)			Wave 7: August 2021 (adjusted)		
Bodily pain		*N*	Mean	SD	*N*	Mean	SE	*N*	Mean	SE	*N*	Mean	SD
	Control	107	64.1	2.7	99	57.8	2.3	88	63.2	3.1	70	64.6	2.51
	Treatment	87	62.0	2.9	79	65.0	2.6	67	62.9	3.6	66	59.1	2.44
	Group	Baseline (December 2018)			Wave 4: February 2020 (adjusted)			Wave 6: February 2021 (adjusted)			Wave 7: August 2021 (adjusted)		
Social functioning		*N*	Mean	SD	*N*	Mean	SE	*N*	Mean	SE	*N*	Mean	SE
	Control	107	62.4	2.8	99	61.2	2.8	88	61.6	3.3	70	69.1	2.9
	Treatment	87	60.8	3.2	78	68.3	3.2	66	65.7	3.7	65	61.5	3.0
	Group	Baseline (December 2018)			Wave 4: February 2020 (adjusted)			Wave 6: February 2021 (adjusted)			Wave 7: August 2021 (adjusted)		
Energy over fatigue		*N*	Mean	SD	*N*	Mean	SE	*N*	Mean	SE	*N*	Mean	SE
	Control	107	46.5	2.2	98	44.0	1.9	88	47.2	2.5	69	47.9	2.4
	Treatment	87	42.4	2.3	79	52.0	2.2	67	50.4	3.0	66	46.9	2.4
	Group	Baseline (December 2018)			Wave 4: February 2020 (adjusted)			Wave 6: February 2021 (adjusted)			Wave 7: August 2021 (adjusted)		
Emotional wellbeing		*N*	Mean	SD	*N*	Mean	SE	*N*	Mean	SE	*N*	Mean	SE
	Control	107	65.7	2.3	98	59.6	2.0	88	62.2	2.6	69	66.0	1.9
	Treatment	87	64.4	2.4	79	67.9	2.2	67	68.2	2.3	66	62.5	1.9
	Group	Baseline (December 2018)			Wave 4: February 2020 (adjusted)			Wave 6: February 2021 (adjusted)			Wave 7: August 2021 (adjusted)		
Role limitations due to emotional health		*N*	Mean	SD	*N*	Mean	SE	*N*	Mean	SE	*N*	Mean	SE
	Control	107	62.9	4.0	99	73.6	4.3	87	59.0	4.7	69	62.9	4.5
	Treatment	87	59.8	4.5	79	58.1	3.8	66	59.6	5.5	62	56.3	4.8
	Group	Baseline (December 2018)			Wave 4: February 2020 (adjusted)			Wave 6: February 2021 (adjusted)			Wave 7: August 2021 (adjusted)		
Role limitations due to physical health		*N*	Mean	SD	*N*	Mean	SE	*N*	Mean	SE	*N*	Mean	SE
	Control	107	62.9	4.0	99	62.1	4.0	88	57.5	4.7	70	57.0	4.4
	Treatment	87	59.8	4.5	79	62.2	4.5	67	59.0	5.4	65	55.5	4.5
	Group	Baseline (December 2018)			Wave 4: February 2020 (adjusted)			Wave 6: February 2021 (adjusted)			Wave 7: August 2021 (adjusted)		
Physical functioning		*N*	Mean	SD	*N*	Mean	SE	*N*	Mean	SE	*N*	Mean	SE
	Control	107	72.1	3.0	99	69.1	1.9	88	70.3	3.2	70	70.1	2.4
	Treatment	87	72.8	2.0	79	75.1	2.1	67	68.9	3.8	66	67.2	2.4

**Table 5:** Analysis of covariance (ANCOVA) of treatment and control SEED participants for SF-36 subscale scores by group

General health	*Model 1: ANCOVA for SF-36 general health subscale baseline (December 2018) through Wave 4 (February 2020)*						
		Type III sum of squares	df	Mean square	*F*	Significance	Partial eta squared
	Treatment	51.35	1	51.35	0.12	0.735	0.00
	Error	78,600.31	176	446.59			
	*R*^2^	0.34					
	*Model 1.1: ANCOVA for SF-36 general health subscale Wave 4 (February 2020) through 6 (February 2021)*						
		Type III sum of squares	df	Mean square	*F*	Significance	Partial eta squared
	Treatment	5.72	1	5.72	0.02	0.888	0.00
	Error	39,171.10	137	285.92			
	*R*^2^	0.62					
	*Model 1.2: ANCOVA for SF-36 general health subscale Wave 6 (February 2021) through 7 (August 2021)*						
		Type III sum of squares	df	Mean square	*F*	Significance	Partial eta squared
	Treatment	0.04	1	0.04	0	0.99	0.00
	Error	34,746.06	130	267.28			
	*R*^2^	0.63					
Bodily pain	*Model 2: ANCOVA for SF-36 bodily pain subscale (December 2018) through Wave 4 (February 2020)*						
		Type III sum of squares	df	Mean square	*F*	Significance	Partial eta squared
	Treatment	2463.10	1	2463.10	4.72	0.031*	0.03
	Error	91,241.82	175	521.38			
	*R*^2^	0.35					
	*Model 2.1: ANCOVA for SF-36 bodily pain subscale Wave 4 (February 2020) through 6 (February 2021)*						
		Type III sum of squares	df	Mean square	*F*	Significance	Partial eta squared
	Treatment	1165.21	1	1165.21	2.56	0.112	0.02
	Error	62,282.42	137	454.62			
	R2	0.47					
	*Model 2.2: ANCOVA for SF-36 bodily pain subscale Wave 6 (February 2021) through 7 (August 2021)*						
		Type III sum of squares	df	mean square	*F*	Significance	Partial eta squared
	Treatment	165.65	1	165.65	0.49	0.485	0.00
	Error	43,899.91	130	337.69			
	*R*^2^	0.61					
Social functioning	*Model 3: ANCOVA for SF-36 social functioning subscale (December 2018) through Wave 4 (February 2020)*						
		Type III sum of squares	df	Mean Ssquare	*F*	Significance	Partial eta squared
	Treatment	2208.77	1	2208.77	2.84	0.094	0.02
	Error	135,571.87	174	779.15			
	*R*^2^	0.15					
	*Model 3.1: ANCOVA for SF-36 social functioning subscale Wave 4 (February 2020) through 6 (February 2021)*						
		Type III sum of squares	df	Mean square	*F*	Significance	Partial eta squared
	Treatment	5.80	1	5.80	0.01	0.925	0
	Error	87,607.57	135	648.95			
	*R*^2^	0.31					
	*Model 3.2: ANCOVA for SF-36 social functioning subscale Wave 6 (February 2021) through 7 (August 2021)*						
		Type III sum of squares	df	Mean square	*F*	Significance	Partial eta squared
	Treatment	1267.92	1	1267.92	2.53	0.114	0.02
	Error	64,634.15	129	501.04			
	*R*^2^	0.44					
Energy over fatigue	*Model 4: ANCOVA for SF-36 energy over fatigue subscale baseline (December 2018) through Wave 4 (February 2020)*						
		Type III sum of squares	df	Mean square	*F*	Significance	Partial eta squared
	Treatment	2774.56	1	2774.56	7.51	0.007**	0.04
	Error	64,324.64	174	369.68			
	*R*^2^	0.21					
	*Model 4.1: ANCOVA for SF-36 energy over fatigue subscale Wave 4 (February 2020) through 6 (February 2021)*						
		Type III sum of squares	df	Mean square	*F*	Significance	Partial eta squared
	Treatment	265.95	1	265.95	0.83	0.363	0.01
	Error	43,463.29	136	319.58			
	*R*^2^	0.44					
	*Model 4.2: ANCOVA for SF-36 energy over fatigue subscale Wave 6 (February 2021) through 7 (August 2021)*						
		Type III sum of squares	df	Mean square	F	Significance	Partial eta squared
	Treatment	26.45	1	26.45	0.11	0.736	0.00
	Error	30,212.10	130	232.40			
	*R*^2^	0.59					
	*R*^2^						
Emotional wellbeing	*Model 5: ANCOVA for SF-36 emotional wellbeing subscale baseline (December 2018) Through Wave 4 (February 2020t*						
		Type III sum of squares	df	Mean square	*F*	Significance	Partial eta squared
	Treatment	3033.10	1	3033.10	7.75	0.006**	0.04
	Error	68,104.25	174	391.40			
	*R*^2^	0.27					
	*Model 5.1: ANCOVA for SF-36 emotional wellbeing subscale Wave 4 (February 2020) through 6 (February 2021)*						
		Type III sum of squares	df	Mean square	*F*	Significance	Partial eta squared
	Treatment	12.81	1	12.81	0.05	0.829	0
	Error	37,252.82	136	273.92			
	R^2^	0.44					
	*Model 5.2: ANCOVA for SF-36 emotional wellbeing subscale Wave 6 (February 2021) through 7 (August 2021)*						
		Type III sum of squares	df	Mean square	*F*	Significance	Partial eta squared
	Treatment	719.72	1	719.72	3.03	0.084	0.02
	Error	30,911.37	130	237.78			
	*R*^2^	0.52					
Role limitations due to emotional health	*Model 6: ANCOVA for SF-36 role limitations-emotional subscale baseline (December 2018) through Wave 4 (February 2020)*						
		Type III sum of squares	df	Mean square	*F*	Significance	Partial eta squared
	Treatment	9930.15	1	9930.15	7.05	0.009**	0.04
	Error	236,559.67	168	1408.09			
	*R*^2^	0.22					
	*Model 6.1: ANCOVA for SF-36 role limitations-emotional subscale Wave 4 (February 2020) through 6 (February 2021)*						
		Type III sum of squares	df	Mean square	*F*	Significance	Partial eta squared
	Treatment	2215.79	1	2215.79	1.43	0.235	0.01
	Error	203,487.48	131	1553.34			
	*R*^2^	0.22					
	*Model 6.2: ANCOVA for SF-36 role limitations-emotional subscale Wave 6 (February 2021) through 7 (August 2021)*						
		Type III sum of squares	df	Mean square	*F*	Significance	Partial eta squared
	Treatment	1.27	1	1.27	0.00	0.973	0.0
	Error	139,333.18	128	1088.54			
	*R*^2^	0.38					
Role limitations due to physical health	*Model 7: ANCOVA for SF-36 role limitations-physical subscale baseline (December 2018) through Wave 4 (February 2020)*						
		Type III sum of squares	df	Mean square	*F*	Significance	Partial eta squared
	Treatment	0.86	1	0.86	0.00	0.981	0.00
	Error	276,236.47	175	1578.49			
	*R*^2^	0.16					
	*Model 7.1: ANCOVA for SF-36 role limitations-physical subscale Wave 4 (February 2020) through 6 (February 2021)*						
		Type III sum of squares	df	Mean square	*F*	Significance	Partial eta squared
	Treatment	143.13	1	143.13	0.11	0.742	0.00
	Error	180,581.17	137	1318.11			
	*R*^2^	0.33					
	*Model 7.2: ANCOVA for SF-36 role limitations-physical subscale Wave 6 (February 2021) through 7 (August 2021)*						
		Type III sum of squares	df	Mean square	*F*	Significance	Partial eta squared
	Treatment	1448.60	1	1448.60	1.37	0.244	0.0
	Error	137,207.13	130	1055.44			
	*R*^2^	0.50					
Physical functioning	*Model 8: ANCOVA for SF-36 physical functioning baseline (December 2018) through Wave 4 (February 2020)*						
		Type III sum of squares	df	Mean square	*F*	Significance	Partial eta squared
	Treatment	1592.64	1	1592.64	4.40	0.037*	0.03
	Error	63,394.48	175	362.25			
	*R*^2^	0.56					
	*Model 8.1: ANCOVA for SF-36 physical functioning subscale Wave 4 (February 2020) through 6 (February 2021)*						
		Type III sum of squares	df	Mean square	*F*	Significance	Partial eta squared
	Treatment	1593.53	1	1593.53	4.49	0.036*	0.01
	Error	48,611.70	137	354.83			
	*R*^2^	0.63					
	*Model 8.2: ANCOVA for SF-36 physical functioning subscale Wave 6 (February 2021) through 7 (August 2021)*						
		Type III sum of squares	df	Mean square	*F*	Significance	Partial eta squared
	Treatment	329.32	1	329.32	0.87	0.352	0.01
	Error	49,094.37	130	377.65			
	*R*^2^	0.62					

Narrative data demonstrated wellbeing patterns that contextualized primary outcomes and explained unexpected secondary outcomes. Early on, participants softened scarcity’s impact by paying bills and meeting basic needs. As volatility smoothed and psychological distress dampened, their time use and pooling behaviors shifted. Pooling references managing scarcity through combining material and immaterial resources across networks. These networks shaped how the $500 spilled into other households and alleviated strain elsewhere. Most spillovers overlapped with food insecurity and unpaid care work for children, older adults, and the medically fragile. Rather than their norm of borrowing food, money, or time for childcare and eldercare from others, they stretched resources across fragile networks. This changed food quality and quantity and assisted with meeting medical needs otherwise missed. During the pandemic, GI initially provided networks ways of reducing exposure through bulk shopping, but as the pandemic deepened, they pre-emptively altered food quality fearing their financial situation would weaken. As Vanessa notes, food is where strain starts saying, “I don’t eat as much. My meals are different. I eat bologna sandwiches and cheerios.”

Financial scarcity also generated time scarcity linked to persistent anxiety and stress which GI dissipated. Many echoed Pam’s words, “I had panic attacks and anxiety…I had to take a pill for it. And I haven’t even touched them in awhile.” When the treatment group crossed from scarcity to stability and psychological distress to wellbeing, they experienced an expansion of time for themselves that Jake described as “normal activities that a lot of people take for granted.” Others linked time expansion to meaningful participation in acts granting “dignity,” including prioritizing relationships, attending social gatherings, reconnecting with family, resuming artistic pursuits long abandoned, and parents able to “breathe and do homework,” host birthdays, and watch “tv with my kids instead of yelling.” These wellbeing trajectories included newfound capacity for goals and control over one’s future by providing the space for people to choose themselves rather than logging additional time in the contingent workforce while struggling to make ends meet. Sarah, like many, described it as a newfound outlook where she could “focus more on myself… To focus on me and get everything I need to be paid in full.”

### Secondary outcomes

During the pre-pandemic year, and holding baseline constant, the treatment group reported a significantly increased capacity to handle an unexpected $400 emergency (*F* = 13.906, *p* =  < 0.001) than did control (Table 6). This effect dissipated between groups in future observations that occurred during the pandemic. To further investigate financial wellbeing, the Financial Wellbeing Scale was added at Wave 5. No significant effect was detected between groups on this scale during the pandemic nor after the withdrawal of the intervention (Table 7). Preliminary reporting noted a substantial increase in full and part-time employment among the treatment group during year one [30]. Trend data in the pre-pandemic year of the experiment show substantial shifts from unemployment to employment (full time, part time, or as a caregiver) from baseline to Wave 4, but were not statistically significant. Further observation during the pandemic, as well as after withdrawal of the intervention did not show significant effects on employment, but did continue to trend toward the treatment group’s continued employment growth compared to control (Table 8).

**Table 6:** Descriptive statistics of treatment and control SEED participants for ability to cover a $400 emergency, February 2019 through August 2021

Wave 2 and covariate adjusted descriptive statistics for $400 emergency question Wave 2, Wave 4, and Wave 7												
Group	Wave 2 (February 2019)			Wave 4: February 2020 (adjusted)			Wave 6: February 2021 (adjusted)			Wave 7: August 2021 (adjusted)		
	N	Mean	SD	N	Mean	SE	N	Mean	SE	N	Mean	SE
Control	115	0.2	0.0	80	0.3	0.1	86	0.4	0.1	67	0.5	0.1
Treatment	103	0.3	0.0	76	0.5	0.1	66	0.5	0.1	64	0.5	0.1
Model 1: Analysis of covariance (ANCOVA) of treatment and control SEED participants for ability to cover a $400 emergency scores by group, Wave 2 (February 2019) through Wave 4 (February 2020)												
	Type III sum of squares	df	Mean square	F	Significance	Partial eta squared						
Treatment	2.80	1	2.80	13.91	< .001	0.08						
Error	30.75	153	0.20									
R^2^	0.16											
Model 2: Analysis of covariance (ANCOVA) of treatment and control SEED participants for ability to cover a $400 emergency scores by group, Wave 4 (February 2020) through 6 (February 2021)												
	Type III sum of squares	df	Mean square	F	Significance	Partial eta squared						
Treatment	0.03	1	0.03	0.15	0.698	0.00						
Error	25.82	131	0.20									
R^2^	0.23											
Model 2: Analysis of covariance (ANCOVA) of treatment and control SEED participants for ability to cover a $400 emergency scores by group, Wave 6 (February 2021) through 7 (August 2021)												
	Type III sum of squares	df	Mean square	F	Significance	Partial eta squared						
Treatment	0.00	1	0.00	0.02	0.884	0						
Error	16.83	126	0.13									
R^2^	0.48

**Table 7:** Descriptive statistics of treatment and control SEED participants for financial wellbeing scale scores, August 2020 through August 2021

*Covariate adjusted descriptive statistics for financial wellbeing scale scores Wave 5, Wave 6, and Wave 7*										
Group	Wave 5: August 2020			Wave 6: February 2021 (adjusted)			Wave 7: August 2021 (adjusted)			
	*N*	Mean	SD	*N*	Mean	SE	*N*	Mean	SE	
Control	80	49.5	1.6	75	48.8	1.7	55	49.9	1.4	
Treatment	68	51.9	1.4	60	50.7	1.6	52	51.0	1.4	
*Model 1: Analysis of covariance (ANCOVA) of treatment and control SEED participants for financial wellbeing scale scores, Wave 5 (August 2020) through Wave 6 (February 2021)*										
	Type III sum of squares	df	Mean square	*F*	Significance	Partial eta squared				
Treatment	0.14	1	0.14	0.00	0.962	0				
Error	6423.46	104	61.76							
*R*^2^	0.70									
*Model 2:Analysis of covariance (ANCOVA) of treatment and control SEED participants for financial wellbeing scale scores, Wave 6 (February 2021) through Wave 7 (August 2021)*										
	Type III sum of squares	df	Mean square	*F*	Significance	Partial eta squared				
Treatment	50.90	1	50.90	0.68	0.410	0.01				
Error	8352.53	112	74.58							
*R*^2^	0.69

**Table 8:** Descriptive statistics of treatment and control SEED participants for employment changes, December 2018 through August 2021

Baseline and covariate adjusted descriptive statistics for employment changes												
Group	Baseline: December 2018			Wave 4: February 2020 (adjusted)			Wave 6: February 2021 (adjusted)			Wave 7: August 2021 (adjusted)		
	N	Mean	SD	N	Mean	SE	N	Mean	SE	N	Mean	SE
Control	137	0.8	0.0	65	0.8	0.0	65	0.7	0.1	44	0.8	0.1
Treatment	71	0.8	0.0	46	0.9	0.1	46	0.8	0.1	39	0.8	0.1
Model 1: ANCOVA for employment changes baseline (December 2018) through Wave 4 (February 2020)												
	Type III sum of squares	df	Mean square	F	Significance	Partial eta squared						
Treatment	0.21	1	0.21	1.80	0.183	0.02						
Error	12.48	108	0.12									
R^2^	0.17											
Model 2: ANCOVA for employment changes Wave 4 (February 2020) through 6 (February 2021)												
	Type III sum of squares	df	Mean square	F	Significance	Partial eta squared						
Treatment	0.03	1	0.03	0.26	0.612	0.00						
Error	10.31	84	0.12									
R^2^	0.35											
Model 3: ANCOVA for employment changes Wave 6 (February 2021) through 7 (August 2021)												
	Type III sum of squares	df	Mean square	F	Significance	Partial eta squared						
Treatment	0.00	1	0.00	0.01	0.909	0.00						
Error	11.24	82	0.14									
R^2^	0.27											

Employment status was shifted from a categorical to binary variable and coded as 1 = eligible for employment and employed (full-time employed, part-time employed, stay-at-home parent or caregiver) or 0 = eligible for employment but not employed (unemployed and looking for work and unemployed and not looking for work). Individuals who indicated they were ineligible for employment due to retirement, disability, or student status were excluded from the analyses

Integrated data revealed an interdependence between agency and risk capacity. When the treatment group stabilized, expansion of time and finances arrived with self-determination and capacity for risk-taking not present prior. GI removed material barriers like childcare funds, transportation, reducing contingent labor, and completing necessary internships or training for applying to positions with unknown results. When one missed paycheck produces eviction or utility loss, it creates material barriers to these small but meaningful risks and the GI altered this. Second, a distinct pattern encompasses “the ability to breathe” and “rethink.” Freedom from scarcity translated into bandwidth that dovetailed with an increase in agency and risk associated with “space” and “breath.” Participants demonstrated how setting alternative pathways requires freedom and the ability to choose risks when outcomes are uncertain. These concepts were inseparable and captured by Kent stating, “you can take so much risk… The only reason I got the internship was because of me taking the risk of having to quit a job before and knowing that I have that money. I could sustain myself until this new opportunity came around, and I was able to take it.” Conversely, “poverty means lack of choice.” This interdependence extended into COVID but presented differently.

COVID shifted risk contours, but some of GI’s power for safeguarding self-determination remained. The $500 permitted judiciousness about COVID and what conditions workers would tolerate for poorly compensated work. Akin to reducing contingent work for pursuing stronger employment, workers avoided COVID exposure by expecting more from their employers when they had a GI floor. Brendan explicitly connected the $500 to what he would endure saying, “I’m just not going to put myself through minimum wage work again.” Then, like others, credited agency with expanding perspective saying, “there’s more to life than just giving money, earning money… there’s memory, there’s culture, there’s art, it all enriches you.”

Agency also exposed a lack of structural support for women and children mirroring national trends. One in ten women resigned during the pandemic, with half crediting school closures, and 47% taking unpaid leave to manage childcare and online education, which are compounded by race and ethnicity [31]. SEED reflects these disparities and complicates the agency GI provides when structural risks limit personal choice and collides with gendered expectations of care work. Ann, who was caring for elderly parents and children, described how the $500 effectively patched holes in the safety net pre-pandemic only to see the power of cash curtailed under the dual burden of covid and care work saying, “You feel like Gumby in a way. You know, you’re just being pulled in so many different physical, and mental, and economic ways… I can’t be stretched any thinner…’how am I gonna survive?” Some possessed more employment freedom, but COVID-induced care work precluded others like June from paid work. June managed remote education for 4 children while caring for her medically fragile father in a small apartment—herself sleeping in the living room and her father sleeping underneath the staircase. While she craved the stability and meaning she enjoyed in management, her level of compensation was not enough to warrant risky costs of COVID while trying to perform unpaid care. For her and others, the floor GI provided allowed her freedom to care for her family, but at the cost of absorbing structurally produced risks when supports for her father and children were eliminated.

Finally, COVID disrupted risk, trust, and agency. At baseline, participants referenced prior experiences with predatory finance that shaped wariness about GI. These memories returned with COVID, prompting comparisons between disinformation in the press and the market. The pandemic reversed or complicated trust-building processes due to confusing public health orders and the lack of agency people felt in vulnerable situations described as life in “The United States of Risk.” These dynamics were further complicated by wildfires which came with competing instructions and exposure to health trouble, along with shifts elsewhere such as methadone clinics sending patients home with more medication than ordinarily allowed or providers canceling appointments. Across all, most either had a pre-existing condition or cared for someone with one putting them at higher risk for COVID and/or breathing trouble from smoke. Thus, they made sense of GI through the lens of pre-existing and emergent vulnerabilities that created new exposures to risk. Subject 665 weighed the risks of procuring food alongside threats posed by smoke and COVID leading to more expensive means like DoorDash when the risk felt too high saying, “I just couldn’t breathe. I just turned around and came home. It was like ‘Nah, canceled.’ And as it is you know with the pandemic, you know you can only really go to the store, and you know go out when you really need stuff, like you know? I’m like, “I, I needed groceries, but uh not that bad I guess.” In this case, his pre-existing health condition was forcing him to pit the need for air against the need for groceries. These repetitive trade-offs ultimately meant he often resorted to expensive food delivery apps which eroded his finances further and echo Ehrenreich’s (2014, p.1) claim that “it is expensive to be poor.”

## Discussion

Integrated analysis indicates GI recipients were rational economic actors, using GI to manage risk by supporting themselves and their networks while weathering the pandemic. There is a causal link between GI and reduced income volatility and improved psychological and physical health, that created opportunities for agency. These results support the counterfactual—as income volatility is associated with negative financial and health outcomes, then guaranteed income does mitigate them.

At the onset of the unprecedent social, economic, and health crisis of the pandemic, the treatment group was overall more financially secure and healthier. There was no significant difference between the treatment and control group on labor—a particularly important finding given the speculation that individuals may become unproductive if given unconditional cash. The significance of those impacts dissipated as the pandemic had critical financial and health impacts across both the treatment and control groups. We note that the trends of a positive trajectory remained higher in the treatment than control group, yet did not reach the level of statistical significance for most measures—possibly due to attrition or simply that the $500 per month was simply not enough to overcome significant structural inequalities that proliferated during the pandemic. In sum, the evidence of the RCT suggests that guaranteed income, under normative economic and health conditions, does calm income volatility and allay financial, emotional, and psychological distress. In atypical conditions, the effects of guaranteed income are inconclusive and worthy of additional investigation. As the withdraw of the intervention and final observation occurred during the pandemic, the lasting effects of guaranteed income are also unknown. As the world returns to normative economic and health conditions, the public health impacts of a national GI could be profound.

These results have limitations. Limited power prevented subgroup analysis and attrition may have impacted the ability to detect effects of the intervention during the pandemic observation period. Generalizability is limited to the population the sample drew from. Some benefits were unable to be preserved, prompting some to withdraw and others to ignore recruitment. This limits findings to those comparatively less fearful of benefits loss. Moreover, attrition in the study could have been differential by outcome variables. However, because those participants did attrite, there is no possibility to test this. These limitations inspire future research that will be undertaken with the SEED data. Notably, future research could investigate long range outcomes on interactions with public systems including safety net programs, incarceration, and education as well as intergenerational impacts. Future research on guaranteed income writ large should focus on differential impacts of dosage and duration on the previously tested outcomes.

When Thomas Paine argued for basic income in 1797 [32], poverty assumptions were cemented in the Protestant work ethic tying dignity to market performance and precluding single women, indigenous people, and people of color from the social contract. American discourse carefully avoids how prior inequality shapes present disparity. This creates pejorative deservedness narratives that shape policy while deterring people from benefits and blaming them for structurally induced positionality [33]. As control group member Jasmine noted, these dynamics likewise hinder collective action saying, “guaranteed income is necessary to stop the war on the poor… how about some class solidarity, we really need that.” When policymakers consider how to best implement and deliver guaranteed income, they must be mindful of how these pejorative discourses manifest materially for intended populations—from privitization and profiteering in service delivery to exclusion based on means testing and other conditions.

Given promising new evidence that could have a nontrivial impact on public health, we must consider which policy pathways GI could follow. As the number of pilots continues growing, a federal waiver is necessary for all safety net benefits to test GI’s impact alongside existing structures. GI should not replace the existing safety net, as the affordable housing crisis and lack of infrastructure for working families threatens economic mobility. Exemptions of GI payments from counting as income have been granted in a handful of locations [33], but most still weigh the benefits of GI against loss of SNAP or TANF. An executive action to waive GI payments would provide a pathway to studying the total impact of unconditional cash.

## Supplementary Information

Below is the link to the electronic supplementary material.

Supplementary file1 (DOC 59 KB)

## References

[1] Basu S (2017). Income volatility: a preventable public health threat. Am J Public Health.

[2] Morduch J, Siwicki J (2017). In and out of poverty: episodic poverty and income volatility in the US financial diaries. Soc Serv Rev.

[3] Elfassy T, Swift SL, Glymour MM (2019). Associations of income volatility with incident cardiovascular disease and all-cause mortality in a US cohort. Circulation.

[4] Prause J, Dooley D, Huh J (2009). Income volatility and psychological depression. Am J Community Psychol.

[5] Grasset L, Glymour MM, Elfassy T (2019). Relation between 20-year income volatility and brain health in midlife. Neurology.

[6] Center on Poverty and Social Policy. CPSP. https://www.povertycenter.columbia.edu/news-internal/2020/COVID-projecting-monthly-poverty/. Accessed 30 Mar 2023.

[7] Addo M, Servon L. Income volatility and healthcare decision-making. https://www.brookings.edu/wp-content/uploads/2021/05/20210506_ServonAddo_IncomeVolatilityHealthcare_Final.pdf. Accessed 31 May 2022.

[8] Mani A, Mullainathan S, Shafir E, Zhao J (2013). Poverty impedes cognitive function. Science.

[9] Shah AK, Mullainathan S, Shafir E (2012). Some consequences of having too little. Science.

[10] Halliday T. Income volatility and health. papers.ssrn.com. Published May 23, 2008. https://papers.ssrn.com/sol3/papers.cfm?abstract_id=1136396. Accessed 6 Mar 2023.

[11] Gunasekara FI, Carter K, Blakely T. Change in income and change in self-rated health: systematic review of studies using repeated measures to control for confounding bias. Social Science & Medicine. 2011;72(2):193–201. 10.1016/j.socscimed.2010.10.029. 10.1016/j.socscimed.2010.10.029 21146277

[12] Sun S, Huang J, Hudson DL, Sherraden M (2021). Cash transfers and health. Annu Rev Public Health.

[13] Forget EL. The Town with no poverty: the health effects of a Canadian guaranteed annual income field experiment. Can Public Policy. 2011;37(3):283–305. 10.3138/cpp.37.3.283.

[14] Aizer A, Eli S, Ferrie J, Lleras-Muney A (2016). The long-run impact of cash transfers to poor families. Am Econ Rev.

[15] Tarasuk V. Implications of a basic income guarantee for household food insecurity series| June 2017. https://proof.utoronto.ca/wp-content/uploads/2017/06/Paper-Tarasuk-BIG-EN-17.06.13-1712.pdf. Accessed 6 Mar 2023.

[16] Costello EJ (2010). Association of family income supplements in adolescence with development of psychiatric and substance use disorders in adulthood among an American Indian population. JAMA.

[17] Widerquist K (2005). A failure to communicate: what (if anything) can we learn from the negative income tax experiments?. J Socio-Econ.

[18] Harvey D. A Brief History of Neoliberalism. OUP Catalogue. Oxford: Oxford University Press; 2005.

[19] Tashakkori A, Teddlie C. SAGE Handbook of Mixed Methods in Social & Behavioral Research. 2nd ed. Thousand Oaks, CA: Sage Publications; 2010. 10.4135/9781506335193.

[20] Castro A, West S, Samra S, Cusack M. Mitigating the loss of health insurance and means tested benefits in an unconditional cash transfer experiment: implementation lessons from Stockton’s guaranteed income pilot. SSM Popul Health. 2020;11:100578. 10.1016/j.ssmph.2020.100578. 10.1016/j.ssmph.2020.100578 PMC7142678 32289072

[21] 36-Item Short Form Survey Instrument (SF-36). www.rand.org. https://www.rand.org/health/surveys_tools/mos/36-item-short-form/survey-instrument.html. Accessed 31 May 2022.

[22] Kessler RC, Andrews G, Colple LJ (2002). Short screening scales to monitor population prevalences and trends in non-specific psychological distress. Psychol Med.

[23] CFPB financial well-being scale: scale development technical report. Consumer Financial Protection Bureau. https://www.consumerfinance.gov/data-research/research-reports/financial-well-being-technical-report/. ‌Accessed 31 May 2022.

[24] Lloro A, Merry E, Brevoort K, et al. Codebook for te 2021 Survey of Household Economics and Decisionmaking. https://www.federalreserve.gov/consumerscommunities/files/SHED_2021codebook.pdf. Accessed 31 May 2022.

[25] Patton MQ. Qualitative research & evaluation methods: integrating theory and practice. 4th ed. Thousand Oaks, CA: Sage Publications, Inc; 2015.

[26] West S, Castro A, Balakrishnan S, Rao K, Tan G. Pre-analysis plan: Stockton economic empowerment demonstration. https://static1.squarespace.com/static/6039d612b17d055cac14070f/t/605029f652a6b53e3dd39044/1615866358804/SEED+Pre-analysis+Plan.pdf. Accessed 30 Mar 2023.

[27] Braun V, Clarke V. Using thematic analysis in psychology. Qual Res Psychol. 2006;3(2):77–101. 10.1191/1478088706qp063oa.

[28] Saldaña J. The coding manual for qualitative researchers. Thousand Oaks: Sage; 2021.

[29] Charmaz K. Constructing grounded theory. 2nd ed. Thousand Oaks: Sage; 2014.

[30] West S, Castro A, Samra S, Coltrera E. Preliminary Analysis: SEED’s First Year. https://static1.squarespace.com/static/6039d612b17d055cac14070f/t/603ef1194c474b329f33c329/1614737690661/SEED_Preliminary+Analysis-SEEDs+First+Year_Final+Report_Individual+Pages+-2.pdf. Accessed 30 Mar 2023.

[31] Ranji U. Women, work, and family during COVID-19: findings from the KFF women’s health survey. KFF. Published March 22, 2021. https://www.kff.org/womens-health-policy/issue-brief/women-work-and-family-during-covid-19-findings-from-the-kff-womens-health-survey/. Accessed 30 Mar 2023.

[32] Paine T, Spence T, Union I, Earthsharing Devon (Network. Agrarian Justice. Earthsharing Devon, 21St March; 2017.

[33] Wollensack H, Neighly M. The benefits cliff and guaranteed income. https://gicp.info/s/Benefits-Fact-SheetJune-2021.pdf. Accessed 6 Mar 2023.

