# Supplementary material for: Impact of Guaranteed Income on Health, Finances, and Agency: Findings from the Stockton Randomized Controlled Trial
Source: J Urban Health. 2023 Apr 10;100(2):227–44. doi: 10.1007/s11524-023-00723-0 (PMC10160253; doi:10.1007/s11524-023-00723-0)
Supplement: Supplementary file 1 — Supplementary file1 (DOC 59 KB) [file 11524_2023_723_MOESM1_ESM.doc]

**
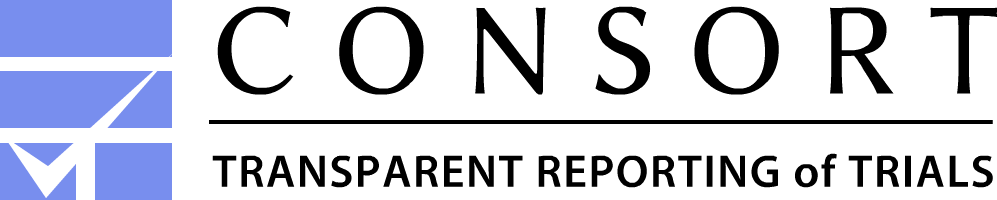
**

**CONSORT 2010 Flow Diagram**

**Allocation**

**Analysis**

**Follow-Up**

**Enrollment**

Assessed for eligibility (n=4,200) )

Excluded (n=3,722)

♦  Did not respond to recruitment mailer (n=3,695)

- Duplicate application (n=27)

Analysed intent to treat (n=198)
♦ No exclusions

Lost to follow-up (give reasons) (n=120)

- Withdrew from study (n=2)
- Unable to contact (n=118)

Allocated to active control, no intervention (n=200)

- Allocated to administrative control, no intervention, no follow up, not reported in this analysis per PAP (n=135)

Lost to follow-up (give reasons) (n=67)

- Withdrew from study, received intervention (n=7)
- Unable to contact (n=60)

Discontinued intervention (give reasons) (n= )

Allocated to treatment (n=131)

♦ Received allocated intervention (n=131)

♦ Did not receive allocated intervention, declined, were replaced from eligible pool (n=12)

Analysed intent to treat (n=110)
♦ Excluded from analysis due to media exposure (n=14)

Randomized (n=478)
